# Psychometric Properties of the German Version of the Health Regulatory Focus Scale

**DOI:** 10.3389/fpsyg.2017.02005

**Published:** 2017-11-14

**Authors:** Bjarne Schmalbach, Roy Spina, Ileana Steffens-Guerra, Gabriele H. Franke, Sören Kliem, Michalis P. Michaelides, Andreas Hinz, Markus Zenger

**Affiliations:** ^1^Department of Psychology, University of Münster, Münster, Germany; ^2^Department of Psychology and Counselling, University of Chichester, Chichester, United Kingdom; ^3^Faculty of Applied Human Studies, University of Applied Sciences Magdeburg-Stendal, Stendal, Germany; ^4^Criminological Research Institute of Lower Saxony, Hannover, Germany; ^5^Department of Psychology, University of Cyprus, Nicosia, Cyprus; ^6^Integrated Research and Treatment Center (IFB) AdiposityDiseases - Behavioral Medicine, Medical Psychology and Medical Sociology, University of Leipzig Medical Center, Leipzig, Germany

**Keywords:** regulatory focus, health, mental health, validation study, short scale, psychometric properties

## Abstract

The Health Regulatory Focus Scale (HRFS) is a short scale which measures an individual's prevention and promotion focus in a health-specific context. The main objective of this study was to examine the psychometric properties of the newly translated German version of the HRFS. Reliability and item characteristics were found to be satisfactory. Validity of both subscales toward other psychological constructs including behavioral approach and avoidance, core self-evaluations, optimism, pessimism, neuroticism, as well as several measures of physical and mental health was shown. In addition, invariance of the measure across age and gender groups was shown. Exploratory as well as confirmatory factor analyses clearly indicated a two-factorial structure with a moderate correlation between the two latent constructs. Differences in health promotion and prevention focus between socio-demographic groups are discussed. The HRFS is found to be a valid and reliable instrument for the assessment of regulatory focus in health-related environments.

## Introduction

Associations between health status and perceived quality of life are well-documented across many socio-demographic variables including age and gender (Carranza Rosenzweig et al., 2004; Zubaran et al., 2008). The World Health Organization (2006) defines health as “complete physical, mental and social well-being and not merely the absence of disease or infirmity” (p. 1). Health should therefore be seen as the avoidance of risks—namely injuries and illnesses—as well as the approach toward general wellness. In order to ensure the efficacy of such psychological and medical programs, it is essential to identify the target population as accurately as possible (Thacker et al., 2006). This will allow for the modification of interventions with regard to specific needs of individuals or groups (Peeling et al., 2010). Therefore, it becomes important to understand how individuals differ in regard to their health behavior and health needs. More specifically, what strategies do different individuals pursue in order to improve their health? Do they approach activities that are beneficial to their health or concentrate on minimizing harmful factors?

Regulatory focus theory deals with approaching and avoiding behavior in a general context (Higgins, 1997). It states that all striving action will be made up by either one or both of the following systems: The *promotion system* focusses on progress and improvement while the *prevention system* centers on the maintenance of the already achieved and avoidance of decline. This means that promotion-oriented individuals will tend to look for advantages and seize opportunities whereas prevention-oriented individuals will lean toward a careful approach and attempt to minimize risks as much as possible (Avnet and Higgins, 2003). How individuals process and use information will be influenced accordingly, depending on an individual's regulatory focus (Pham and Avnet, 2004). Furthermore, an individual can easily be primed into a specific regulatory focus state by suitable stimuli (Pham and Chang, 2010). Such chronic and temporary phenomena both play an important role in explaining human behavior and thinking (Keller and Bless, 2006). The regulatory focus systems are rooted in specific neural components, as indicated by neural correlates that have been identified, including an activation of the amygdala, the anterior cingulate cortex, and the extrastriate cortex (Cunningham et al., 2005). Additionally, promotion focus is related to an activation of the right prefrontal cortex while prevention focus correlates with an activation of the left prefrontal cortex (Amodio et al., 2004).

It is important to distinguish regulatory focus systems from the evidently similar approach-avoidance system (Elliot and Thrash, 2002). Studies have shown, however, that approach-avoidance motivation and regulatory focus are indeed moderately correlated (Mooradian et al., 2008; Summerville and Roese, 2008). However, Higgins (1997) maintains that both systems operate independent of one another. Additionally, Molden et al. (2008) show how prevention and promotion focus are best conceptualized as two independent factors. Their 2 × 2 model demonstrates how it is easily possible for a person to have an approach motivation while at the same time being prevention-oriented, rather than being promotion-oriented. In the same way, an avoidance motivation can be combined with both regulatory foci. For example, exercising, which is a common approach-focused health behavior, can be associated with either promotion focus (improving one's heart function) or prevention focus (keeping one's heart function from declining).

The concept of regulatory focus therefore makes it possible to examine individuals regarding processes of goal setting and attainment, which has proven to be useful in various domains. For example, in marketing and consumer psychology it allows for the adaptation of advertisements to the particular focus of the recipient (Mowle et al., 2014). Conversely, advertisements can be generated specifically to suggest a certain regulatory focus to recipients and influence decision processes by such means (Micu and Chowdhury, 2010). In health services we are also confronted with such patterns of behavior and thought. As mentioned in the beginning, health behavior consists of components of approach as well as avoidance behavior. To this end, theory of regulatory focus appears to be suited for the characterization of health behavior and health cognitions.

In reality however, there are several critical limitations. Bearden et al. (2001) argue that a general measure—as both aforementioned questionnaires of regulatory focus clearly are—can never be as valid and accurate as a domain-specific one. Correspondingly, there are a number of health-specific instruments which have been adapted from general constructs to fit into a health context (Gomez et al., 2013). Additionally, Hooker and Kaus (1992) were able to show that learned health behavior does not necessarily translate into actual behavior and that a majority of individual health beliefs and values are first formed in midlife. This means it is entirely possible for a person to be promotion-oriented in general but have a prevention focus when it comes to health matters, especially when one considers the controversial nature of health issues (Wang et al., 2010). Furthermore, Gomez et al. (2013) argue that studies which have been conducted on regulatory focus and health (van Kleef et al., 2005; Vartanian et al., 2006; Uskul et al., 2008; Schokker et al., 2010) may have been unable to show hypothesized relationships because of the utilized measurement's missing focus on health issues.

Gomez et al. (2013) developed the *Health Regulatory Focus Scale* (HRFS) to allow the measurement of regulatory focus in health-specific contexts. It assesses health promotion focus as well as health prevention focus. Concordant with earlier research (Pham and Higgins, 2005; Avnet and Higgins, 2006) health promotion items deal with the seeking and seizing of opportunities to improve one's health while health prevention items capture an individual's attempts to avert dangers to their health. A two-factor solution was found to be the most suitable for the HRFS. Extracted variance for each dimension exceeded 50%, allowing Gomez et al. to conclude that convergent validity of the questionnaire is confirmed. Varying correlations between health prevention focus and health promotion focus were found in several study samples, ranging between *r* = 0.16 and *r* = 0.57. To further explore the HRFS's validity, Gomez et al. examined the relationships between health regulatory focus and a number of constructs. They found moderate to weak positive correlations between health promotion focus and optimism (Grant and Higgins, 2003) and between health prevention focus and neuroticism (Amodio et al., 2004; Otonari et al., 2012). Moreover, they showed that higher levels of health promotion focus as well as lower levels of health prevention focus can predict subjective health status (Elliot and Sheldon, 1998; Jung et al., 2013).

The objectives of the present study are (a) to investigate the dimensionality of the newly conceived German version of the HRFS (b) to evaluate its psychometric properties; (c) and to build upon the work of Gomez et al. (2013) by continuing to explore the validity of the scale in terms of associations between health regulatory focus and related constructs, as well as differences in health regulatory focus based on sociodemographic variables. We expect the following correlations: As mentioned above, moderate correlations between promotion focus and behavioral approach as well as between prevention focus and behavioral inhibition are known (Carver et al., 2000; Elliot and Thrash, 2002; Summerville and Roese, 2008), and thus we expect the same associations for health regulatory focus. The connection between pessimism and general prevention focus has been known for some time (Peterson et al., 1988; Grant and Higgins, 2003). Furthermore, Grossardt et al. (2009) could predict all-cause mortality using pessimism as well as anxious and depressive personality traits, which equals focusing on the negative, as an individual with a health prevention focus would. Therefore, the present study assumes the same relationship for pessimism and health prevention focus, and a negative one with optimism. We would expect the opposite pattern for health promotion focus. In light of neuroticism being a part of the *Core Self-Evaluations*, we hypothesize that there is an association of positive self-evaluations with health promotion focus as well as of negative self-evaluations with health prevention focus (Ferris et al., 2013). Finally, we expect negative correlations between health outcomes and health prevention focus, and positive relationships for health promotion focus.

## Materials and methods

### Participants and procedures

The sample of the study was collected by means of the online survey tool SoSciSurvey (Leiner, 2015). Data acquisition began in December 2015, after approval by the ethics commission of the University of Applied Sciences Magdeburg-Stendal (AZ-3973-51), and ended in February 2016. The study was advertised on several social networks and bulletin boards, of which some but not all had a health, sports, and/or nutrition background. Participants were educated about the general topic of the study and gave their informed consent in accordance with the Declaration of Helsinki, before they could start the questionnaire. The total number of participants who first gave consent is *N* = 1,173, of which *n* = 292 (25%) aborted the survey before answering all questions. Participants who aborted the survey after giving at least part of their socio-demographic information but before completing any of the other presented questionnaires (*n* = 250; 21%) differed significantly in terms of their reported gender (*U* = 84356.50, *p* < 0.001), males being more likely to abort, and education (*U* = 86094.00, *p* = 0.008), with participants of lower education being more likely to abort, from those who completed additional questionnaires. Those participants who quit immediately after answering the HRFS (*M* = 29.23, *SD* = 11.131) did not differ significantly from those participants who continued (*M* = 29.40, *SD* = 10.801) on any variables collected but were included nonetheless to avoid a selection bias, as the difference in age distributions was very close to being significant (*U* = 12580.00, *p* = 0.055, *d* = 0.01). Because of the nature of the design of the online survey, participants answered all HRFS items or none at all; there was no missing data. Individuals who were too young to take part in the study—namely under the age of 16 years—were excluded. Thus, the used sample consisted of *N* = 923 participants.

Participants who were included in the analysis had a mean age of around 30 years (*M* = 29.59; *SD* = 10.86) with a range from 16 to 70 years. Detailed characteristics of the sample are presented in Table 1. Compared to the population averages, which were obtained from the Federal Statistical Office of Germany (2013), the sample was relatively young. Also, women are over- and men under-represented. Participants reported a higher level of education than expected in the general population, with more than seventy percent having achieved a university entrance qualification, compared to approximately thirty percent in the general population. Household net income was lower than in the general population, which could also be due to a higher ratio of singles and young people in the sample. Finally, in comparison to the general population, participants were more likely to be students or apprentices, and less likely to be working, unemployed, staying at home, or retired.

**Table 1:** Socio-demographic characteristics of the full study sample as well as means and standard deviations for the health regulatory focus subscales.

	***N***	**%**	**Health promotion focus^a^**	**Health prevention focus^a^**
**GENDER**				
Female	748	81.0	4.43 (1.24)	3.16 (1.60)
Male	170	18.4	4.02 (1.42)	2.97 (1.55)
Other	5	0.5	4.20 (1.37)	3.50 (1.00)
**AGE (YEARS)**				
≤20	141	15.3	4.41 (1.25)	3.40 (1.68)
21–30	486	52.7	4.31 (1.26)	3.15 (1.55)
31–40	147	15.9	4.30 (1.25)	3.09 (1.61)
>40	149	16.1	4.48 (1.41)	2.83 (1.58)
**FAMILY STATUS**				
Single	505	54.7	4.33 (1.26)	3.19 (1.58)
Committed relationship	219	23.7	4.39 (1.25)	3.29 (1.65)
Married	143	15.4	4.46 (1.35)	2.83 (1.52)
Separated	11	1.1	4.06 (1.07)	3.45 (1.68)
Divorced	38	4.1	4.12 (1.57)	2.55 (1.47)
Widowed	7	0.7	4.43 (0.80)	2.07 (0.93)
**EDUCATION**				
Pupil	26	2.8	4.52 (1.58)	3.29 (1.66)
≤8 years	24	2.6	4.66 (1.75)	3.19 (1.91)
9–11 years	120	13.0	4.41 (1.40)	3.29 (1.60)
12–13 years	412	44.6	4.33 (1.24)	3.24 (1.62)
University	341	36.9	4.33 (1.22)	2.92 (1.51)
**EMPLOYMENT STATUS**				
Working full time	276	29.9	4.36 (1.31)	3.02 (1.62)
Working part time	123	13.3	4.41 (1.26)	3.13 (1.73)
Student/Apprentice	449	48.6	4.34 (1.25)	3.20 (1.53)
Unemployed	38	4.1	4.15 (1.27)	3.16 (1.68)
Homemaker	18	2.0	4.52 (1.52)	2.50 (1.41)
Retired	19	2.1	4.32 (1.52)	3.42 (1.56)
**HOUSEHOLD NET INCOME**				
<1,000 €	337	36.5	4.38 (1.24)	3.13 (1.55)
1,000–1,999 €	227	24.6	4.51 (1.23)	3.34 (1.63)
≥2,000 €	291	31.5	4.22 (1.31)	2.91 (1.54)
No answer	68	7.4	4.27 (1.46)	3.34 (1.72)

a *Group means and standard deviations are presented as M (SD)*.

### Measures

#### Health regulatory focus scale (HRFS)

Gomez et al. (2013) developed the HRFS to measure promotion and prevention focus in health-specific contexts. The scale consists of eight items in total, five of which measure health promotion focus and three of which deal with health prevention focus. Answer options are presented on a 7-point-scale and range from (1) “strongly disagree” to (7) “strongly agree.” Taking the average of the items in question yields the respective scale score. Internal consistency for the scale is reported by Gomez et al. as α = 0.88 for health promotion focus and α = 0.77 for health prevention focus. The English version items were translated by two professional translators independent of one another. Afterward, they had to reach a consensus on a singular version for each item, which was then back-translated by two native speakers and compared with the original. Reliability coefficients are reported in the results of the study. Both language versions are displayed in the **Table 3**.

#### Behavioral inhibition/approach system scale (BIS/BAS)

The BIS/BAS (Carver and White, 1994; Strobel et al., 2001) are used to measure motivational processes of approach and avoidance. Twenty items are split among four scales (*BIS, BAS-Drive, BAS-Fun Seeking, BAS-Reward Responsiveness*) in addition to four filler items. Scale values are calculated by averaging item scores after inverting two of them. Strobel et al. (2001) reported the internal consistency of the *BIS* scale as α = 0.78, and the *BAS* scale as a whole as α = 0.81.

#### Big five inventory-10 (BFI-10)

In order to efficiently measure personality, the BFI-10 was used (Rammstedt and John, 2007), which is a short scale for assessing the *Big Five* personality dimensions *Openness, Conscientiousness, Extraversion, Agreeableness*, and *Neuroticism*. One item per scale needs to be inverted before further calculations. The mean of the two items which make up each scale then represents the respective scale score. Retest-reliability coefficients for the German version are *r*_*tt*_ = 0.78 for *Openness, r*_*tt*_ = 0.83 for *Conscientiousness, r*_*tt*_ = 0.66 for *Agreeableness, r*_*tt*_ = 0.87 for *Extraversion*, and *r*_*tt*_ = 0.71 for Neuroticism, according to Rammstedt and John.

#### Core self-evaluations scale (CSES)

The CSES (Judge et al., 2003; Zenger et al., 2015) measures a higher order personality construct which includes facets of self-esteem, locus of control, neuroticism, and self-efficacy, using 12 items. Zenger et al. (2015) recommended treating the positively-worded and the negatively-worded items as loading on two distinct factors for the German version. Therefore, the two respective scale scores are obtained by taking the mean of the items in question. Internal consistency according to Zenger et al. (2015) lies between α = 0.81 and 0.86.

#### Life orientation test–revised (LOT-R)

The LOT-R (Scheier et al., 1994; Glaesmer et al., 2008) measures optimism and pessimism using three items each. In addition, the scale uses four filler items. Although Scheier and Carver (1985) propose a one-factor solution with optimism and pessimism as two extremes of the same continuum, a two-factor interpretation is to be preferred, particularly for the German version (Herzberg et al., 2006). Both scale scores are obtained by adding up the respective items. Internal consistency of optimism is reported by Glaesmer et al. (2012) as α = 0.70 and pessimism as α = 0.74.

#### Patient health questionnaire-4 (PHQ-4)

The PHQ-4 (Kroenke et al., 2009) is a brief screening tool for symptoms of depression and anxiety of four items. It asks participants to what extent they suffered from said symptoms during the last 2 weeks. By summing up the individual item scores one receives the scale score. Löwe et al. (2010) report α = 0.82 for the scale.

#### Somatic symptom scale-8 (SSS-8)

To measure somatic symptoms, the SSS-8 was applied (Gierk et al., 2014). It consists of eight items, that ask for experienced somatic stress in the last 7 days. The total score results from the addition of all individual items. Gierk et al. (2014) report the internal consistency of the scale as α = 0.81.

#### Self-reported health status

From the EuroQol-5D (Brooks et al., 2003) a visual analog scale (VAS), ranging from (0) “worst imaginable health status” to (100) “best imaginable health status,” was used to measure the self-reported health status of participants.

### Statistical analyses

The majority of statistical calculations was conducted using IBM SPSS Statistics 20. The confirmatory factor analysis (CFA) was performed in IBM AMOS 20. All correlations are reported using the Pearson product-moment correlation coefficient. Tests of significance use an α level of 0.05 unless otherwise noted. Properties of all used scales and all items of the HRFS were determined, namely means and deviations as well as item difficulty and item-total correlations for HRFS items. The item difficulty index signifies how well an item differentiates between different groups of participants. An index of 0 means that all participants chose the lowest possible answer, while an index of 1 means all participants chose the highest possible option. For an item to add diagnostic value to the scale, a difficulty index between 0.20 and 0.80 is considered desirable. Those same scales and items were tested for normality of distribution by calculating skewness and kurtosis. The assumptions of sphericity and sampling adequacy were controlled. We conducted exploratory factor analysis (EFA) to determine the ideal number of factors. Subsequently, we used CFA to test how well the model suggested by theory and the EFA fit the empirical data. We randomly split our sample into two subsamples of approximately equal size (*n*_EFA_ = 444; *n*_CFA_ = 479). Those subsamples did not differ significantly in terms of age, gender, and HRFS item scores. To further solidify the results of the EFA, the minimum average partial (MAP) test (Velicer, 1976) and parallel analysis (PA) (Horn, 1965) were utilized. The MAP test uses average squared partial correlations of the items to determine the ideal number of factors. PA calculates eigenvalues based on randomly generated correlation matrices which have the same number of variables and cases as the original raw data and tests them for significant differences from the empirically found ones. For those two tests, syntaxes from O'connor (2000) were used. For the CFA, covariance matrices and the maximum likelihood method were used. To judge the fit of the calculated models, the following commonly used indices were applied. First, minimum discrepancy divided by degrees of freedom (CMIN/DF) should be as low as possible (Schermelleh-Engel et al., 2003) and preferably lower than 5 (Hu and Bentler, 1999). The comparative fit index (CFI) and the Tucker–Lewis Index (TLI) should be larger than 0.95 to indicate good fit and larger than 0.90 to be recognized as acceptable, whereas the standardized root mean square residual (SRMR) should be lower than 0.08 (Hu and Bentler, 1998). The root mean square error of approximation (RMSEA) and its 90% confidence interval should be lower than 0.10 to be considered barely acceptable fit, lower than 0.08 to be considered fair fit, and lower than 0.06 to be considered good fit (MacCallum et al., 1996; Hu and Bentler, 1999). Finally, the Bayesian Information Criterion (BIC) was used for the comparison of models, which favors a lower value (Schermelleh-Engel et al., 2003).

Measurement invariance was tested using multiple-group analysis in a two-step process. First, the configural model (without constraints) was compared to the metric model (constraining unstandardized item loadings to be equal across groups). Secondly, the metric model and the scalar model (constraining unstandardized item loadings and intercepts across groups) were compared. For this comparison, the differences in CFI and gamma hat (Steiger, 1989) between models were examined, as is recommended by previous research (Cheung and Rensvold, 2002; Milfont and Fischer, 2010). Differences in χ^2^ were considered and reported as well. However,—because of χ^2^'s sensitivity to sample size—emphasis was put on ΔCFI and Δgamma hat. Sociodemographic groups were tested for differences in mean values using ANOVA, excluding groups that did not have at least 20 members. Requirements of normal distribution and equal variances were checked and could be confirmed. To counteract the accumulation of α error probability, a significance level of 0.01 was employed in these comparisons. *Post-hoc* tests were conducted using Tukey's HSD. Effect sizes are reported as Cohen's *d* with >0.2 being a small, >0.5 being a medium, and >0.8 being a large effect (Cohen, 1992).

## Results

### Item characteristics and reliability

Skewness and kurtosis of the investigated items and scales was found to be lower than the commonly used cutoff values (absolute value of skewness < 1; absolute value of kurtosis < 3; Bulmer, 1979; Byrne, 2010). Thus, a normal distribution of item and scale scores can be assumed. Item difficulty indices of the HRFS were found to be between 0.42 and 0.71, while corrected item-whole correlations were between 0.54 and 0.71 with the exception of item 5, which had a comparatively low correlation of 0.36 with its total score, justifying its exclusion based on the cutoff value of 0.50 (Hair et al., 2010). Exact values for item and scale characteristics are reported in Table 2. Cronbach's α of the HRFS was 0.86 for the health promotion scale and 0.72 for the health prevention scale. An inclusion of item 5 would have led to a decline of internal consistency to 0.66. The inter-correlation of the scales was *r*_(921)_ = 0.20, *p* < 0.001.

**Table 2:** Characteristics of the HRFS items using the full study sample.

**Item/Scale**	***M* (*SD*)**	**Skewness**	**Kurtosis**	***P***	***r_*it*_***
HRFS 1	4.98 (1.55)	−0.63	−0.22	0.71	0.60
HRFS 3	4.50 (1.59)	−0.34	−0.59	0.64	0.64
HRFS 4	4.11 (1.73)	−0.04	−0.90	0.59	0.72
HRFS 6	3.83 (1.68)	0.07	−0.98	0.55	0.72
HRFS 8	3.78 (1.60)	0.10	−0.79	0.54	0.71
H-Promotion scale	4.24 (1.31)	−0.12	−0.50		
HRFS 2	3.34 (1.85)	0.41	−1.02	0.48	0.54
HRFS 5	4.47 (1.72)	−0.34	−0.82	0.64	0.36
HRFS 7	2.92 (1.75)	0.77	−0.47	0.42	0.56
H-Prevention scale	3.13 (1.59)	0.61	−0.52		

*P, difficulty index; r_it_, item-total correlation. Scale means and standard deviations exclude item 5*.

### Factor structure

**Figure 1:**
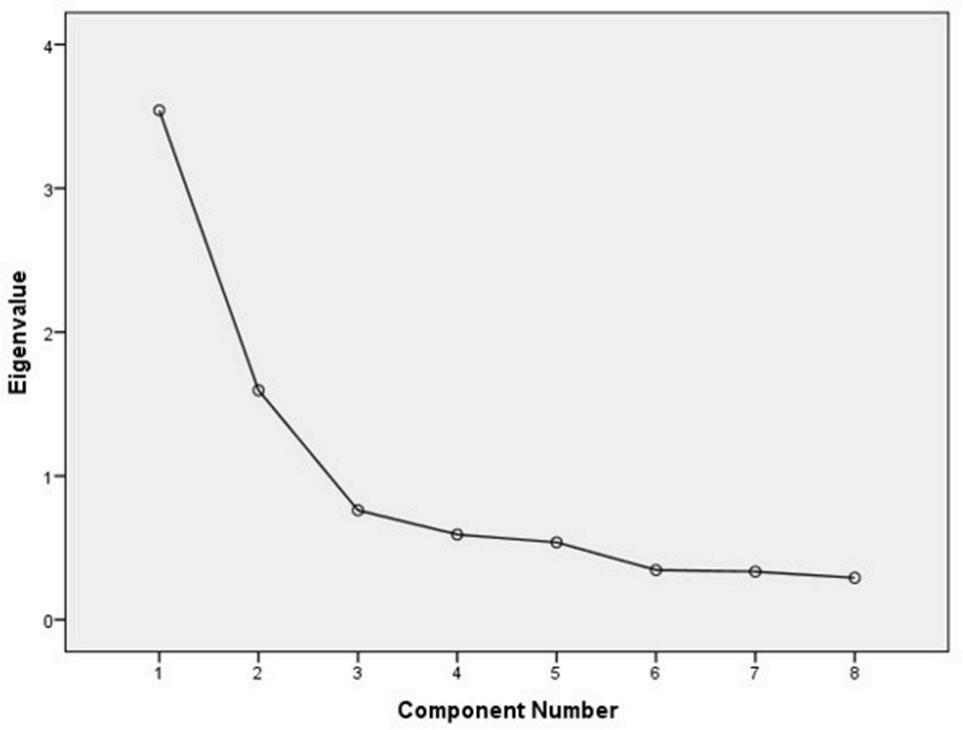
Scree plot of the EFA for the HRFS.

The EFA was conducted with the first subsample using a principal component analysis (PCA) with Varimax rotation and Kaiser normalization. It suggested a two-factorial solution with eigenvalues of 3.28 and 1.86, explaining 41% and ~23% of variance. As can be seen in Table 3, factor loadings showed strong associations between all items and their respective factor. No item exhibited factor loadings smaller than 0.7 on its hypothesized factor with the exception of item 5. The screeplot also indicated a distinct decline of explained variance after two factors (see Figure 1). The average partial correlations between variables were lowest when assuming one factor in the MAP test. In the PA, however, both empirically found eigenvalues of factors one and two were larger than to be expected with a 95% margin of error. Thus, the MAP test showed evidence for a one-factorial solution, whereas Horn's PA further consolidated the findings of the PCA by also suggesting two factors (see Table 4 for MAP and PA results).

**Table 3:** Factor loadings of all HRFS items in the EFA.

**Item**	**German**	**English**	**Promotion**	**Prevention**
HRFS 1	Ich zögere nicht, Neues auszuprobieren, wenn ich der Meinung bin, dass ich dadurch meine Gesundheit verbessern kann.	I do not hesitate to embrace new experiences if I think they can improve my health.	0.75	−0.05
HRFS 2	Ich denke häufig über die gesundheitlichen Probleme nach, die ich eines Tages haben könnte.	I frequently think about the health problems I may have in the future.	−0.04	0.86
HRFS 3	Wenn ich ein Ziel in Hinblick auf meine Gesundheit erreicht habe, spornt mich das an, mich noch mehr zu steigern.	If I succeed in reaching a health goal, this motivates me to go further.	0.76	0.13
HRFS 4	Ich genieße es, mich um meine Gesundheit zu kümmern.	I think that taking care of my health is pleasurable.	0.81	0.05
HRFS 5	Wenn ich mein Gesundheitsverhalten ändere, dann tue ich es, um mich vor Krankheiten zu schützen.	When I implement a health behavior, it's because I want to protect myself from getting sick.	0.40	0.54
HRFS 6	Ich betrachte mich als jemanden, der sein Möglichstes tut, um seine Gesundheit zu verbessern.	I see myself as someone who does my utmost to improve my health.	0.82	0.17
HRFS 7	Ich mache mir oft Sorgen, Fehler zu machen, die meine Gesundheit beeinträchtigen könnten.	I often worry about mistakes I could make concerning my health.	0.09	0.86
HRFS 8	Wenn ich eine gute Gelegenheit sehe, um meine Gesundheit zu verbessern, ergreife ich sie sofort.	If I see a good opportunity to improve my health, I take advantage of it right away.	0.81	0.22

**Table 4:** Results of the minimum average partial test and parallel analysis.

**Factors**	**MAP test**	**PA eigenvalues**	
	**Average squared partial correlations**	**Raw data**	**Random data^a^**
0	0.154		
1	0.055	3.542	1.267
2	0.065	1.596	1.177
3	0.110	0.761	1.110

a *The random data represents the upper limit of the 95% confidence interval of the eigenvalue distribution of 1,000 random data sets*.

Based on the contradictory findings of the EFA and the results Gomez et al. (2013) reported for the factorial structure of the original version of the HRFS, the CFA was conducted with subsample 2 testing a one-factor as well as a two-factor model. Table 5 shows the model fit indices of this solution and all other considered models. Since the model fit was not acceptable and item 5 exhibited exceptionally high modification indices as well as moderate loadings on both factors—as evidenced by the low item-total correlation—a model that excludes item 5 was considered and tested. This new model showed a sizeable improvement over both previously examined ones. Health promotion focus items loaded on one factor with loading between 0.66 and 0.81, while items measuring health prevention focus loaded on the other factor with loadings of 0.61 and 0.90. Individual factor loadigns are reported in Table 6. The correlation of both latent constructs was *r*_(921)_ = 0.28, *p* < 0.001.

**Table 5:** Model fit indices of the calculated factor models.

**Model**	**χ^2^(*df*)**	**CMIN/DF**	**CFI**	**SRMR**	**RMSEA [90% CI]**	**TLI**	**BIC**
One-factor	278.02 (20)	13.90	0.821	0.103	0.164 (0.147, 0.182)	0.749	376.76
Two-factor	145.00 (19)	7.63	0.912	0.089	0.118 (0.100, 0.136)	0.871	249.93
Two-factor B^a^	57.48 (13)	4.42	0.965	0.037	0.085 (0.063, 0.108)	0.943	150.06

*CMIN/DF, minimum discrepancy divided by degrees of freedom; CFI, comparative fit index; SRMR, standardized root mean square residual; RMSEA, root mean square error of approximation including 90% confidence interval, TLI, Tucker-Lewis index; BIC, Bayesian Information Criterion*.

a *The Two-factor model B excludes HRFS item 5*.

**Table 6:** Standardized CFA factor loadings as per Model B.

**Item**	**Promotion**	**Prevention**
HRFS 1	0.66	
HRFS 2		0.68
HRFS 3	0.80	
HRFS 4	0.81	
HRFS 6	0.79	
HRFS 7		0.61
HRFS 8	0.91	

*All loadings were significant (p < 0.001)*.

Subsequently, invariance across gender and age was analyzed using the entire sample. The results of this analysis are displayed in Table 7. Since a few groups presented Heywood cases under certain model constraints (Dillon et al., 1987; Gerbing and Anderson, 1987), the original two-factor model, including item 5, was utilized to test for measurement invariance. The differences in CFI and gamma hat between models did not exceed 0.01. Therefore, scalar invariance could be shown for males and females as well as for different age groups.

**Table 7:** Fit indices for the multigroup analysis.

**Model**	**χ^2^(*df*)**	**Δχ^2^**	**Δ*p***	**CFI**	**ΔCFI**	**GH**	**ΔGH**
**GENDER MULTIGROUP ANALYSIS**							
Configural invariance	307.47 (38)			0.901		0.931	
Metric invariance	319.00 (44)	11.54	0.073	0.899	0.002	0.9300	0.001
Scalar invariance	344.20 (55)	25.12	0.009	0.894	0.005	0.926	0.004
**AGE MULTIGROUP ANALYSIS**							
Configural invariance	350.83 (76)			0.903		0.930	
Metric invariance	369.15 (94)	18.32	0.435	0.903	0.000	0.931	0.001
Scalar invariance	422.27 (127)	53.12	0.015	0.895	0.008	0.926	0.005

*CFI, comparative fit index; GH, gamma hat*.

### Validity

Correlations between both subscales—excluding item 5—of the HRFS and health-related measures were calculated and are presented in Table 8. The health promotion scale showed moderate correlations with the BAS, the optimism subscale of the LOT-R, as well as the positive CSES. On the other hand, the health prevention scale had the strongest associations with negative CSES, the BIS, psychological symptoms as well as neuroticism. Further connections to somatic symptoms, overall health, and also optimism and pessimism were found.

**Table 8:** Means, standard deviations (in parentheses), reliabilities and correlations with the HRFS along with upper and lower bounds of the 95% confidence intervals (in parentheses) for the employed validation measures.

	***M* (*SD*)**	**α**	**Health promotion focus**	**Health prevention focus**
BIS (Inhibition)	2.91 (0.62)	0.83	0.02 [−0.04; 0.08]	0.40^*^ [0.34; 0.45]
BAS (Activation)	3.02 (0.40)	0.78	0.36^*^ [0.30; 0.41]	−0.06 [−0.13; 0.01]
CSES positive	3.87 (0.64)	0.88	0.23^*^ [0.17; 0.29]	−0.31^*^ [−0.37; −0.25]
CSES negative	2.81 (1.04)	0.81	−0.07 [0.14; 0.0]	0.41^*^ [0.35; 0.46]
Neuroticism	3.01 (1.04)	0.67	−0.01 [−0.08; 0.06]	0.36^*^ [0.30; 0.42]
LOT-Optimism	10.76 (2.53)	0.74	0.20^*^ [0.13; 0.26]	−0.31^*^ [−0.25; −0.37]
LOT-Pessimism	7.22 (2.61)	0.78	−0.06 [−0.13; 0.01]	0.32^*^ [0.26; 0.38]
PHQ-4	7.30 (2.97)	0.85	−0.05 [−0.12; 0.02]	0.39^*^ [0.33; 0.45]
SSS-8	15.58 (5.21)	0.76	−0.03 [−0.10; 0.04]	0.37^*^ [0.31; 0.43]
Health VAS	76.33 (17.43)	–	0.12^*^ [0.05; 0.19]	−0.27^*^ [−0.33; −0.21]

* *Denotes significant correlations (p < 0.05)*.

### Differences based on socio-demographic variables

Means and standard deviations of all compared groups can be found in Table 1. Women were found to be significantly more health promotion-oriented than men, *t*_(230.41)_ = 3.46, *p* = 0.001, *d* = 0.32, while for health prevention focus there was no such difference, *t*_(916)_ = 1.371, *p* = 0.171, *d* = 0.12. With regard to different age groups (<21; 21–30; 31–40; and >40), results of an ANOVA indicated no significant differences for health prevention focus, *F*_(3, 919)_ = 3.15, *p* = 0.024, η^2^_*p*_ = 0.010, nor for health promotion focus, *F*_(3, 919)_ = 0.82, *p* = 0.486, η^2^_*p*_ = 0.002. Between groups of education level there were no significant differences in health promotion focus, *F*_(4, 918)_ = 0.58, *p* = 0.679, η^2^_*p*_ = 0.003, or health prevention focus, *F*_(4, 918)_ = 2.35, *p* = 0.053, η^2^_*p*_ = 0.010. No differences were statistically significant when considering employment status for either health promotion focus, *F*_(3, 882)_ = 0.41, *p* = 0.743, η^2^_*p*_ = 0.001, or health prevention focus, *F*_(3, 882)_ = 0.78, *p* = 0.504, η^2^_*p*_ = 0.003. Groups of different family status showed significant differences when comparing health prevention focus, *F*_(3, 896)_ = 4.68, *p* = 0.003, η^2^_*p*_ = 0.02, but not for health promotion focus, *F*_(3, 896)_ = 0.74, *p* = 0.531, η^2^_*p*_ = 0.002. Finally, no significant differences were found for health promotion focus, *F*_(3, 919)_ = 2.28, *p* = 0.078, η^2^_*p*_ = 0.007, or for health prevention focus, *F*_(3, 919)_ = 3.63, *p* = 0.013, η^2^_*p*_ = 0.011, when comparing groups of household net income. The *post-hoc* analysis did not reveal significant differences for any of the tested comparisons. Effect sizes hardly ever exceed *d* = 0.20 and did not exceed *d* = 0.30, with the exception of the comparison of divorced participants with singles (*d* = 0.41) and participants in committed relationships (*d* = 0.49), where divorced participants showed lower mean health prevention focus than the compared group by trend.

## Discussion

The main aim of the present study was to investigate psychometric properties of the newly translated German version of the HRFS. The dimensionality of the German HRFS corresponds well with the model that was proposed by Gomez et al. (2013) for the original version as well as with the basic regulatory focus theory. This is to say, a two-factor solution is to be preferred, with one of those factors being health promotion focus and the other health prevention focus. This was concluded based on the results of the PCA and the PA as well as the findings of the CFA. A unidimensional solution was considered because of the inadequacy of the original two-factor model in the CFA. However,—as expected based on the results of the EFA—it showed a relatively bad model fit. This is in line with past research on regulatory focus. Promotion and prevention focus have always been conceptualized as two individual factors, not just as two sides of the same coin (Higgins, 1998). Therefore, we excluded item 5, which loaded ambiguously on both latent constructs with factor loadings of around 0.40 to 0.50. The resulting two-factor solution had adequate to good model fit indices. The RMSEA can be considered barely adequate. However, it is suspected that the relatively high value found is due to the small number of manifest variables and the resulting low complexity of the model, which can inflate RMSEA (Kenny and McCoach, 2003; Fan and Sivo, 2007). Inter-correlation of the latent factors was moderate (*r* = 0.28) further verifying the validity of the proposed solution. Metric and scalar invariance could be shown for this instrument with respect to age as well as gender. This means that mean scores of these subgroups can be compared in a statistical manner.

Reliability coefficients were found to be acceptable for both scales (Tavakol and Dennick, 2011). Both coefficients are comparable to what has been found for the original version of the scales. The coefficient for the health prevention focus scale was on the lower spectrum of acceptability although this is most likely due to the shortness of the scale. Other statistical parameters—such as kurtosis and skewness of items and scales as well as difficulty index and item-total correlation were found to be satisfactory. Both scales were shown to correlate moderately with several major psychological constructs. Namely, health promotion focus showed connections to behavioral activation, positive self-evaluation, and optimism. A weak positive correlation with subjective health status was shown as well. Health prevention focus, on the other hand, is associated with behavioral inhibition, negative self-evaluations, neuroticism, pessimism, an absence of optimism, as well as psychological, somatic, and subjective health. These findings largely confirm the expectations we formulated beforehand. Research has been aware of the moderate to strong correlation between regulatory focus and behavioral inhibition for a while now (Summerville and Roese, 2008). The present study delivers another replication of this result and thus reaffirms the need for disentanglement of these two motivational and behavioral constructs. Positive self-evaluations, optimism and pessimism correlated with the HRFS as predicted. Health promotion—striving for good health—was associated with higher optimism. Health prevention, however, showed moderate correlations to lower optimism and higher pessimism. This is in line with our predictions, as individuals with high health prevention concentrate on maintaining a status quo and averting negative outcomes which naturally leads to worrying and focusing on the negative. The overarching personality construct in this relation is neuroticism, as per the Big Five. Individuals high in neuroticism worry a lot, are anxious and fearful. All of these descriptions could also describe a person high in health prevention focus. A moderately high correlation between health prevention—but not health promotion—and the BFI-10 neuroticism scale, confirmed this expectation. The moderate correlations with the CSES deliver further evidence for this line of argument.

The low correlations of health promotion focus with health outcomes are somewhat surprising, but again, demonstrate the two-dimensional nature of regulatory focus. Seeking health gains seems to represent a small part of the overall health puzzle. However, not worrying about losing health status—an individual high in health prevention focus typically would—seems to be more predictive of health status, as evidenced by markedly lower psychological and symptomatic symptoms as well as subjective health status for those with low health prevention focus. These correlations demonstrate how health promotion and prevention focus can explain behavioral and cognitive processes in health-specific contexts. Therefore, the nomological validity of both health regulatory focus constructs could be shown.

We found significant differences in health regulatory focus between groups of several socio-demographic variables. Most interestingly, we found that women are significantly more health-promotion-oriented than men, while there was no difference between genders in health prevention focus. This difference in health regulatory focus could help explain the well-documented disparities in health status and health behaviors between genders and answer some unanswered questions (Kandrack et al., 1991; Dawson et al., 2007; Regitz-Zagrosek, 2012).

The HRFS can allow for a better understanding of the impact that health promotion as well as health prevention focus can have on an individual's response to treatments or health programs. This can, in turn, allow researchers and practitioners to tailor interventions to specific needs of recipients.

### Limitations

Some limitations to this study have to be discussed. First, the acquired sample cannot be considered representative of the German population. Men as well as individuals over the age of 50 are under-represented in our sample in comparison to the general population. Like Gomez et al. (2013) this study is therefore unable to provide any norm data for the measure and recommends an establishment of age and gender norms by further research. Since only a single percentage of the study sample was older than 60, scalar invariance should not be assumed for individuals over this age, and should be tested in further research. Secondly, the analysis of measurement invariance was conducted using the two-factor model which includes item 5 in order to avoid Heywood cases for a few of the constrained models. As the accepted model, consisting of 7 items, is identical to the one that was tested with the exception of the omission of one item, measurement invariance can be accepted. It is still recommended that a study with representative sampling verifies these results.

## Conclusion

To summarize, the HRFS's psychometric properties were found to be good and the assumption of a two-factorial structure showed a good model fit. The questionnaire works equally well for males and females and also for several age groups. It is well suited for an application as a screening tool in health-related contexts such as consumer, personnel, and clinical psychology because of its high validity with regard to constructs like self-evaluations, optimism, pessimism, neuroticism, behavioral approach, and avoidance, as well as mental and physical health status.

## Author contributions

All listed authors have made substantial contributions to the present research in one way or another. BS, MZ, RS, and IS-G contributed to conceptualization and design of the study as well as writing of the manuscript. GF and MM contributed to the data collection and analysis as well as writing of the manuscript. AH and SK contributed to the discussion of the results and writing of the manuscript. All authors agree to be accountable for the content of the work.

### Conflict of interest statement

The authors declare that the research was conducted in the absence of any commercial or financial relationships that could be construed as a potential conflict of interest.
